# Combined Naïve Bayesian, Chemical Fingerprints and Molecular Docking Classifiers to Model and Predict Androgen Receptor Binding Data for Environmentally- and Health-Sensitive Substances

**DOI:** 10.3390/ijms22136695

**Published:** 2021-06-22

**Authors:** Alfonso T. García-Sosa, Uko Maran

**Affiliations:** Institute of Chemistry, University of Tartu, Ravila 14a, 50411 Tartu, Estonia

**Keywords:** androgen receptor, bayesian, multivariate logistic regression, chemical fingerprints, ecfp, docking, toxicity, human, chimp, rat

## Abstract

Many chemicals that enter the environment, food chain, and the human body can disrupt androgen-dependent pathways and mimic hormones and therefore, may be responsible for multiple diseases from reproductive to tumor. Thus, modeling and predicting androgen receptor activity is an important area of research. The aim of the current study was to find a method or combination of methods to predict compounds that can bind to and/or disrupt the androgen receptor, and thereby guide decision making and further analysis. A stepwise procedure proceeded from analysis of protein structures from human, chimp, and rat, followed by docking and subsequent ligand, and statistics based techniques that improved classification gradually. The best methods used multivariate logistic regression of combinations of chimpanzee protein structural docking scores, extended connectivity fingerprints, and naïve Bayesians of known binders and non-binders. Combination or consensus methods included data from a variety of procedures to improve the final model accuracy.

## 1. Introduction

Concern has been rising due to the fact that many environmental factors can modulate the androgen receptor (AR) pathway: agricultural and industrial chemicals, pharmacological drugs and chemotherapeutics, aging, hyperthermia, and chronic infection, alcohol, tobacco, and other drugs [1,2,3,4]. These chemical agents enter the waterways, food chain, and affect other environments. In vivo rodent models are one way to try to elucidate the exact roles of AR in reproduction and the molecular mechanism of AR modulation in reproductive health [1,2,3,4]. Modulation of the AR has multiple biological effects on species in the environment, on health, and diseases. These include important roles in the development and maintenance of reproductive [5], musculoskeletal [6,7], cardiovascular [8,9,10], immune [11,12], nervous [13,14,15], and hematopoietic [16,17] systems. The AR has also been shown to be associated with the development of prostate [18], breast [19,20], bladder [21], liver [22], kidney [23], and lung [24] tumors [25]. Chemicals that mimic hormones can cause abnormalities and undesirable effects in the hormonal system, which in turn can lead to many of the aforementioned diseases. However, the number of synthesized and commercially produced chemicals is constantly increasing, resulting in more of them reaching the environment, the food chain, and eventually the human body. The compounds of most concern are those that may disrupt hormone-receptor binding in the body, or prevent other interactions that are responsible for metabolism, transport, or synthesis of substances, such as endocrine disrupting chemicals (e.g., disrupting the hormonal system) [5,26]. For example, AR binding compounds, or those that interfere with androgen-dependent pathways, can be responsible for increased infertility and decreased sperm counts [27], prostate cancer, testicular dysgenesis syndrome, among others [28,29].

AR pathway interactions may or may not be due to ligand binding on the androgen receptor [30]. The former, ligand-dependent, are in turn divided into two: the androgen molecule/AR pathway event involves DNA (so-called genomic); or where interaction with DNA does not occur (so-called non-genomic) [30]. These AR-activity related interactions can be modeled with a variety of computational approaches [31]. Different techniques can be used to predict the binding of compounds to a receptor, or to infer the binding or activity of a compound. If a protein structure is available, structure-based methods may be used [32,33,34]. Androgen receptor binding has been modeled for toxicology, but also for drug design [32]. Ligand-based methods that use statistical approaches are an alternative for modeling androgen receptor binding without using the structure of the protein, mostly QSAR methods, mainly dealing with specific series of compounds, but also with general series of compounds [31,35,36,37,38,39]. Even if there is a wealth of data on the androgen receptor, it is generally recognized that this is difficult system to model [31], as well as expensive and difficult to assay in vivo, in human, and other model systems.

Modeling opportunities were made available recently via the US Environmental Protection Agency (EPA) Collaborative Modeling Project for Androgen Receptor Activity (CoMPARA) [40]. The CoMPARA project used data from the integrated experimental and computational approach that combined data from 11 ToxCast and Tox21 in vitro high throughput screening (HTS) assays measuring activity at multiple points along the AR pathway: receptor-binding, coregulator recruitment, chromatin-binding of the mature transcription factor, and gene transcription [41]. Within the CoMPARA project, different modeling approaches were used by the consortium of 25 collaborating research teams to evaluate the androgenic potential of compounds, and finally, the prediction results and modelling methods were combined into a consensus [42]. A similar collaborative project was carried out previously to predict compounds with potential estrogenic activity in the Collaborative Estrogen Receptor Activity Prediction Project (CERAPP) [43].

The aim of the present study was to find a method or combination of methods, including consensus modeling, to allow predicting compounds that may bind to the important AR and/or disrupt androgenic pathways, and through this pathway may cause undesired and dangerous health outcomes. These predictions, in turn, can guide decision making and further analysis of compounds. The consensus combinations are employed for molecular docking, chemical fingerprints, naïve Bayesian, and logistic regression methods. Predictions of the final model of this study were originally submitted to the CoMPARA project as one of the three modeling methods of our research group, but were not included into the overall consensus model as during the course of the CoMPARA project, a limit was set for the submissions by one research group.

## 2. Methods

### 2.1. Data Sets and Molecular Structures

The dataset of active, i.e., binding compounds (*n* = 205, composed of both agonists and antagonists), and inactive (i.e., non-binding, *n* = 1480) compounds was obtained from the CoMPARA project website [40,41], and named training set (*n* = 1685, Table S1). A validation data set (*n* = 20, Kleinstreuer et al. [41]) with AR reference compounds included was used, that contained indication of their binding status. An evaluation data set (*n* = 3882, Table S2) was also provided by the CoMPARA project organizers [42] and was processed and scored with the best model trained with the training set. The status for each binding compound was reported as “Binding” in the SDF file (ToxCast_AR_Binding-2016-11-17.sdf). We considered the field “hitcall” in the SDF file that divided the compounds into “Active” and “Inactive” in order to test the procedure. This gave *n*_active_ = 453, *n*_inactive_ = 3429. All chemical structures were used as the QSAR ready SMILES provided within the CoMPARA project; ‘QSAR ready’ means that salts were converted into neutral form, counterions were removed, tautomers were normalized, etc. (detailed description about datasets and their assembly are provided in reference [42]). Minimized 3D structures of the ligands were also prepared by the CoMPARA organizers.

### 2.2. Molecular Docking

The data provided by the CoMPARA organizers contained data including in vitro compound assays on chimp, human, and rat androgen receptors [41]. In the present work we were more interested in grouping the actives as binders, irrespective if they were either agonist or antagonist, as both classes would presumably bind to the AR. The inactive compounds (organizer definition ‘inactive’ was Activity concentration >800 μM) were taken to be non-binders. Docking was performed using Glide XP v. 2017, as contained in the Schrodinger suite [44], with conditions as described previously, [34] with the main difference of docking being performed in the orthosteric site of AR using 15 Å inner box and 40 Å outer search boxes.

### 2.3. Protein Structures

The protein structure for the androgen receptor for chimp (*Pan troglodytes*), human (*Homo sapiens*), and rat (*Rattus norwegicus*) species were downloaded from the PDB [45], with structure codes (resolution) 1t7r (1.4 Å), 3v49 (1.7 Å), and 3g0w (1.95 Å), respectively. These structures were selected based on the availability of a protein-ligand complex, the highest possible X-ray crystal structure resolution, and the completeness of amino acid residue sequence. The best crystal structure among these was selected as the one that gave the best separation of known binders and non-binders. Structure 1t7r contains the AR in complex with 5-alpha-dihydrotestosterone at a resolution of 1.4 Ångströms.

### 2.4. Characterization and Comparison of Ligands

Chemical fingerprints were generated using Extended Chemical Fingerprints (ECFP), a circular fingerprint as encoded by Instant JChem [46]. Distances between chemical fingerprints were calculated by Tanimoto (a.k.a. Jaccard, *T*) coefficient for the case of a binary fingerprint (bit string), according to Equation (1), where N_A_ and N_B_ are the number of bits set in the bit strings of molecules *A* and *B*, respectively, and N*_A_*_&*B*_ is the number of bits that are set in both.

(1) $$T\left(A,B\right)=\frac{N_{A\&B}}{N_{A}+N_{B}-N_{A\&B}}$$

The dissimilarity or distance between molecules is calculated according to Equation (2), where *T*(*A*, *B*) is the Tanimoto coefficient for molecules *A* and *B*.

(2) D(A,B)=1−T(A,B)

### 2.5. Naïve Bayesians

Bayesian classifiers were calculated according to Equation (3), where *μ* is the mean, *σ* is the standard variation, and *x* is the independent variable, in this case the docking scores [47].

(3) $$P\left(x\right)=\frac{1}{\sqrt{2\pi \sigma^{2}}}e^{-\frac{{\left(x-\mu \right)}^{2}}{2\pi \sigma^{2}}}$$

The probabilities are calculated for both binders and non-binders and their ratio for the values calculated for a new compound determines their classification into either group, i.e., if a probability for a compound was higher for the binding group than for the non-binding group, the compound was classified as a binder and vice versa.

### 2.6. Multivariate Logistic Regression

Multivariate logistical regression [48] was used according to Equation (4), where *P_cmpd_* is the probability of a compound of belonging to class 1 (classified as binding), or class 0 (coded non-binding), based on the variables *X*_1…n_ that are properties of the compound and their coefficients α_1…N_.

(4) $$P_{cmpd}=\frac{e^{\left(\beta +\alpha_{1}X_{1}+\ldots \;\alpha_{n}X_{n}\right)}}{1+e^{\left(\beta +\alpha_{1}X_{1}+\ldots \;\alpha_{n}X_{n}\right)}}$$

The linear form of Equation (4) (logit(*Y*)) can have infinitely large or small values for the dependent variable, so instead of ordinary least squares, maximum likelihood techniques are used to maximize the value of the log likelihood (LL) function, which indicates how likely it is to obtain the observed values of *Y*, given the values of the independent variables and the parameters *β*, α_1_, *…*, α*_n_*.

### 2.7. Performance Analysis

The confusion matrix is usually defined as the collection of four fields: true positives (*TP*), true negatives (*TN*), false positives (*FP*), and false negatives (*FN*). Using these values, common measures can be calculated that allow to evaluate the quality of a prediction, such as specificity (*SP*): *TN*/(*TN* + *FP*), sensitivity (*SE*): *TP*/(*TP* + *FN*), accuracy (*Acc.*): (*TP* + *TN*)/(*TP* + *FP* + *FN* + *TN*), and Matthews correlation coefficient (*MCC*). The *MCC* was calculated for all procedures according to Equation (5).

(5) $$MCC=\frac{TP\cdot TN-FP\cdot FN}{\sqrt{\left(TN+FN\right)\cdot \left(TN+FP\right)\cdot \left(TP+FN\right)\cdot \left(TP+FP\right)}}$$

For more detailed analysis, we added several other measures: the probability that a chemical predicted as a binder is actually a binder (*PPV*, Equation (6)), the probability that a chemical predicted as a nonbinder is actually a nonbinder (*NPV*, Equation (7)), positive (+*LR*, Equation (8)) and negative (−*LR*, Equation (9)) likelihood ratios [49], and modified correct classification rate, giving higher scores for models with optimal balance between *SE* and *SP* (*BCR*, Equation (10)) [50].

(6) $$PPV=\frac{TP}{TP+FP}$$

(7) $$NPV=\frac{TN}{TN+FN}$$

(8) $$+LR=\frac{SE}{1-SP}$$

(9) $$-LR=\frac{1-SE}{SP}$$

(10) $$BCR=\frac{SE+SP}{2}\times \left(1-\left|\;SE-SP\right|\;\right)$$

### 2.8. Availability of Best Model

The numerical raw data and best classification model is provided in the QSAR Data Bank format [51] and uploaded to the QsarDB repository [52,53]. A digital object identifier (DOI) is assigned for the model and data [54].

## 3. Results

### 3.1. Androgen Receptor from Chimp as Model Protein

**Figure 1:**
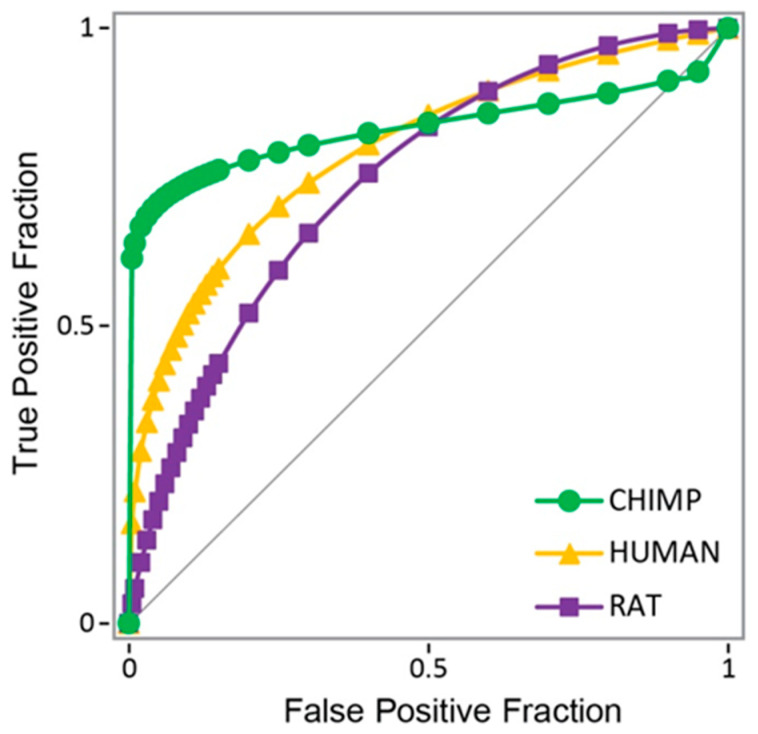
Receiver-operator curves for docking to the human (in yellow), rat (in purple), and chimp (in green) androgen receptor as compared to a random pick (diagonal line). Chimp AUC = 0.832; human AUC = 0.797; and rat AUC = 0.744.

The self-docking of the known binder dihydrotestosterone into structure 1t7r gave a strong docking score of −11.91 kcal/mol. The root-mean-square deviation (RMSD) of atom positions between the docked pose and the initial position of the co-crystallized ligand was 0.32 Å, indicating a good fit and small deviation from the crystal structure. Another known binder, testosterone, also scored a strong docking score of −10.32 kcal/mol. To widen the window for the predictions, we decided heuristically to use a (relatively strong) threshold value of −7 kcal/mol as this would correspond approximately to ligand submicromolar K*_d_* values. After docking the known binders and known non-binders to the three different protein structures from the different species, the results showed best agreement with experimental values (binding status) using the chimp protein structure, rather than using human or rat protein structures in molecular docking, or a combination of the three (Figure 1). Receiver-operator curves and area under the curve (AUC) values provided further validation, giving AUC values of 0.832 for chimp, 0.797 for human, and 0.744 for rat AR.

**Figure 2:**
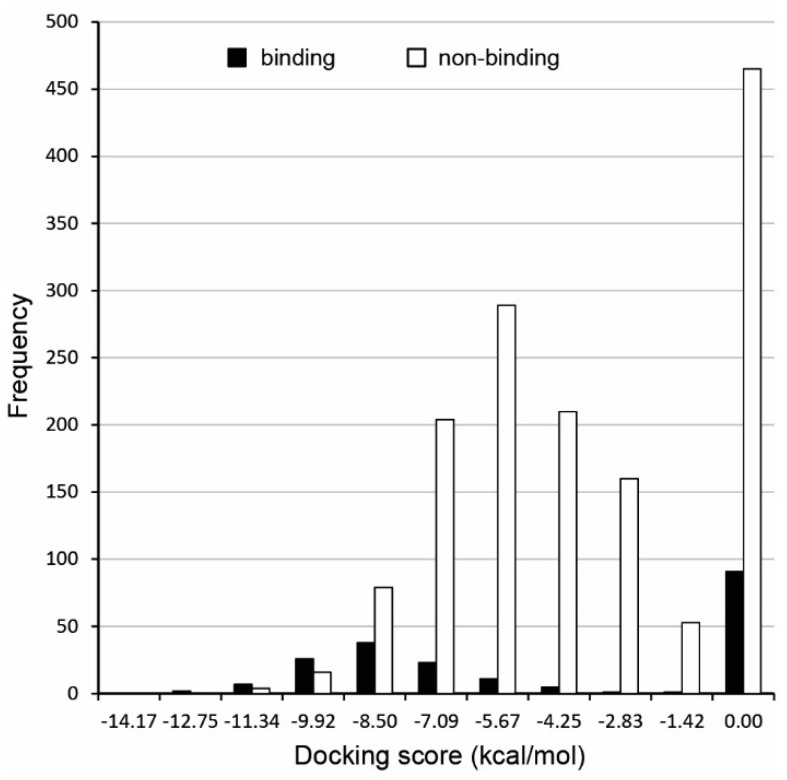
Distributions of docking scores (in kcal/mol) for binding (agonists plus antagonists) and non-binding compounds for the training set against chimp protein.

The chimp docking results distribution was analyzed for both binding and non-binding compounds in the training set (Figure 2), showing that around half of the compounds for both binding and non-binding compounds had a docking score of zero, while the other half was indeed separated, with binding compounds having values distributed over deeper docking scores. This good resolution for compounds with a non-zero docking score prompted to search for a further way to separate the compounds that did not resolve well, i.e., those binding and non-binding compounds that had a docking score of zero.

### 3.2. Consensus Methods for Best Performance

**Figure 3:**
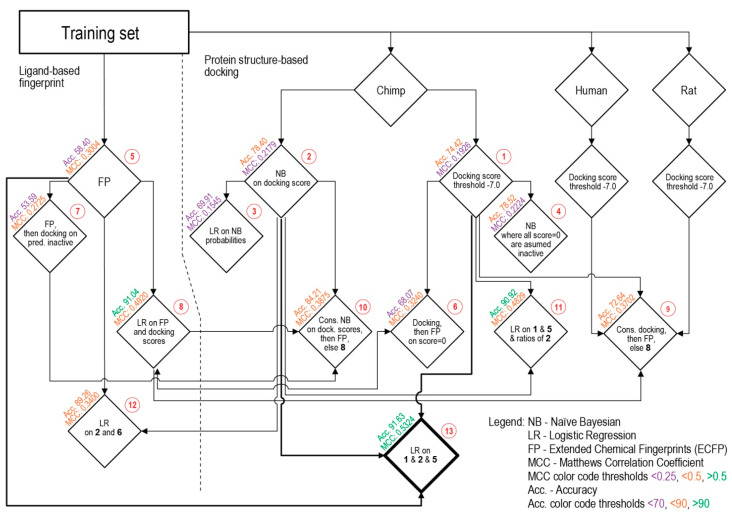
Flowchart of the different Procedures (**1**–**13**, see Section 3.2 in text) applied on the training set.

Subsequently, various methods and combinations thereof were applied to separate the binding and non-binding compounds (Figure 3). For this, several different metrics were checked for each procedure, with the Acc. and MCC as the primary metrics to guide the improvement of the procedures. The range of MCC is from −1, total disagreement between prediction and observation, to +1, a perfect prediction. Different procedures applied to the training set and their sequential relationship are schematically viewed in Figure 3, where horizontal levels from top to bottom show the complexity of combinations and path towards the improvement of the Acc. and MCC values.

A first attempt at separation used a threshold on the docking scores in order to classify compounds into binding and non-binding. Different values were tested, with the best being a threshold score value of −7 kcal/mol. Compounds with scores above this level were classified as non-binders, and scores below this level were classified as binders (Figure 3: Procedure **1**). Despite the fact that the Acc. value is relatively good, the MCC value is the lowest of all the tested Procedures (Table 1).

Naïve Bayesian classifiers were constructed with the docking scores of both active (binding) and inactive (non-binding) compounds, resulting in mean values of *μ* = −8.91 and −5.97 kcal/mol, and standard deviations (SD) = 1.94 and 2.01, respectively. The results of using only this classifier on its own are shown in Table 1: Procedure **2**.

Procedure **3** was similar to Procedure **2**, using a univariate logistic regression on the naïve Bayesian classifier probabilities. Procedure **4** was a modification of the Bayesian classifier, in which the docking score was used as in the first procedure, reducing the number of false positives by assuming zeroes were non-binding compounds. Both procedures did not improve the MCC values, while giving reduced Acc. values.

The following, Procedure **5**, was ligand-based and involved calculating the distance between ECFPs for a compound and the known binders and non-binders. The smaller the distance between the ECFPs and each group, either the average distance towards known binders or average distance to known non-binders, would indicate the similarity of a compound to either group. This procedure resulted in an improved separation and therefore improved MCC at 0.3, while Acc. decreased.

Docking scores and ECFPs were used together for Procedure **6**. Here, the threshold of −7 kcal/mol was used first on the docking scores, and the compounds that registered 0 kcal/mol were then separated using ECFPs. This combination had again the effect of improving the separation and thus, the Acc. and MCC values. Procedure **7** was similar, but was used in the inverted order, i.e., if the ECFP predicted a non-binder, then the docking score threshold was used. The latter procedure was not as effective as the former, giving a lower MCC and the lowest Acc. among Procedures.

For Procedure **8**, a logistic regression was used on the docking scores and difference between ECFP distances to binders and non-binders. This improved the separation and provided the increase in MCC to 0.492 and the second best Acc. among the procedures. Procedure **9**, used a sequence such as in Procedure **6**, first docking scores, then ECFP, and then, if a zero was still obtained, used the logistic regression in Procedure **8**. Neither this, nor Procedure **10** were better than Procedure **8**. Procedure **10** was analogous to procedure **7**, adding the logistic regression from **8** to the sequence.

Procedure **11** used a new logistic regression on the docking scores, ECFP differences, and ratio of Bayesian predicted classifications. This resulted in better separation than **10**, an MCC to 0.4829 and third best Acc. Procedure **12** was similar to **11**, but employed the Bayesian averages instead of the ratio. However, **11** and **12** performed similarly.

Out of all the combinations, a multivariate logistical model emerged as the best performing (Procedures **8** and **11**). The best equation obtained was Procedure **13**, represented as Equation (11) (http://dx.doi.org/10.15152/QDB.235, accessed on 21 June 2021).

(11) $$P_{cmpd}=\frac{e}{1+e^{Y}}=\frac{1}{1+e^{-Y}}$$

$$Y=26.169\;–\;0.0175*\mathrm{ChimpDockScore}\;-98.582{*\mathrm{avgD}}_{\mathrm{Act}}+66.953{*\mathrm{avgD}}_{\mathrm{Inact}}\;+3.584{*\;P}_{\mathrm{Act}\_\mathrm{dockChimp}}\;–\;8.594{*P}_{\mathrm{Inact}}\;\;$$

where

$$Y=26.169\;–\;0.0175*\mathrm{ChimpDockScore}\;-98.582{*\mathrm{avgD}}_{\mathrm{Act}}+66.953\;{*\mathrm{avgD}}_{\mathrm{Inact}}+3.584{*\;P}_{\mathrm{Act}\_\mathrm{dockChimp}}\;–\;8.594{*P}_{\mathrm{Inact}}\;\;$$

This final model includes five descriptors: the docking score for the compound with chimp protein (ChimpDockScore), the average of the distances to the known active (toxic) chemicals (avgD_Act_), the average of the distances to the known inactive (non-toxic) chemicals (avgD_Inact_), the Bayesian probability value for the compound according to the distribution of known actives (toxic) compounds towards the chimp protein (P_Act_dockChimp_), and the Bayesian probability value for the compound according to the distribution of known inactives (non-toxic) compounds towards the chimp protein (P_Inact_).

For Equation (11), Procedure **13** (http://dx.doi.org/10.15152/QDB.235), the best indicator of performance was recorded by a LogLikelihood (LL) value of −407.619. The values for true positives (TP), true negatives (TN), false positives (FP), and false negatives (FN) were: 75, 1469, 11, and 130, respectively. SP (true negative rate) = 99.26%, SE (true positive rate) = 36.59%, Accuracy = 91.63, the first and the last being the highest among tested procedures. The MCC gave a value of 0.5324, which is better than random and the best result for MCC obtained from the several options tried. Removal of 15 compounds referred to as likely false positives [55] gave a slightly better value of MCC = 0.5364. This procedure resulted also in the highest Accuracy value.

In more detailed analysis, NPV, PPV, +LR, −LR, and BCR statistics showed a slightly different view of the models (Table 2). In computational toxicology, one wishes to predict potentially harmful chemicals, minimizing FNs. This implies a classification model is usable even if it gives a high rate of FPs (low PPVs) but a low number of FNs (high NPVs) [56]. With this criterion, Procedure **8** (logistic regression on docking scores and ECFPs) gives the best model, with one of the lowest PPVs of 19.92, and the highest NPV of 98.07.

Positive (+LR) and negative (−LR) likelihoods ratios and balanced classification rate (BCR) are considered to be independent of the data distribution within the training set (noticeably unbalanced) [56]. By these measures, Procedure **8** again has the best, i.e., lowest, −LR value of 0.1420. On the other hand (admittedly less important in computational toxicology, since the objective is to minimize FNs), the best (highest) +LR value of 49.22 belongs to Procedure **5** (ECFPs). Finally, Procedure **10** (consensus Bayesians and ECFP, else **8**) had the highest BCR value of 0.6805. Triscuizzi et al. using a different procedure reported comparable values of +LR = 14.33 at SE = 0.25 for crystal structure 2am9; −LR of 0.38 at SE = 0.75 for crystal structure 2pnu; BCR of 0.62 at SE = 0.75 for 2pnu [56].

### 3.3. Validation of the Best Model

Using the best procedure, the AR pathway in vitro reference compounds presented in Kleinstreuer et al. [41] were used to further validate the model after recording a high docking score for the co-crystallized ligand. The results of the predictions on compounds that had verified experimental data as agonist and antagonist (i.e., without NA values), or that had only one strong or moderate value with the other being NA, are presented in Table 3.

The results in Table 3 on the reference compounds show a good balance of 13 successful predictions versus seven mispredictions. If the moderate/weak compounds bisphenol A, linuron, flutamide, and prochloraz are classified as weak instead of moderate, then the balance of correct to incorrect predictions becomes 15 versus 5, respectively (75%). It is interesting to note that the most successful procedure combined structure-based values: docking to the chimp protein; ligand-based values: distances between extended connectivity fingerprints; and statistical comparisons: naïve Bayesian classifiers. This may reflect the need to use a variety of methods for a complex dataset as the one provided in the CoMPARA project.

An evaluation set provided by the organizers was also used. Results showed reasonably good Acc. of 0.88 and lower MCC values than for the training set, with an MCC of 0.1676 (TP = 36, TN = 3395, FP = 34, FN = 418; SP = 0.99, SE = 0.079) for Procedure **13**, and a slightly better MCC = 0.2036 and lower Acc. of 0.75 (TP = 217, TN = 2713, FP = 716, FN = 235) for Procedure **5**. These MCC values are still higher than random that would correspond to MCC = 0, lower than those obtained for the training set, yet appropriate for an evaluation set that was nearly twice the size of the training set and also highly unbalanced. They are comparable to the MCC obtained using support vector machines (SVM) in a different study by other groups on the same evaluation set [57]. Comparison of MCC values also clearly shows that MCC is not sufficient as the only measure for classification measurement on imbalanced datasets [58]. Procedure **10** has a BCR of 0.68, which is comparable to that obtained on the same evaluation set by a different group using SVM [57]. The Acc. values for Procedures **13** and **5** are comparable to those of Manganelli et al. using SVM, artificial neural networks (ANN), decision trees (DT), SARpy1 (fragment SAR [59]) SARpy2 [59], consensus models [57]. In parallel with this study, we developed a model using a random forest (RF) algorithm for balanced data sets, which did not use molecular docking data and were simpler in design than the current best model and gave the evaluation data set an Acc. of 0.78 (binders model only) [60]. A later development of the present study, the analysis of a balanced data set with deep neural networks (DNN), gave improved Acc. of 0.91 (MCC = 0.4685) [61].

## 4. Discussion

For the protein structural information part of this study, the best crystal structure for these purposes was employed using the available information at the time (see below). This is distinct from the approach of Trisciuzzi et al. [56] who used GOLD software to dock ligand decoys on nine protein structures, separating decoys from known binders and studying their applicability domain, choosing structure 2pnu (resolution of 1.65 Ångströms) instead. The assessment in the present study was done rather on the basis of the higher resolution of the crystal structure, amino acid sequence, and complex availability, and we chose structure 1t7r for the chimp AR, instead. In the present work, this was the crystal structure that best separated binders from non-binders among the CoMPARA data. Comparing results, both approaches are comparable in their active recall rate. Different measures such as MCC, BCR, +LR, and –LR give positive results for Procedures **13**, **8**, and **10**. The consensus model for Procedure **13** is also available for the use at the QsarDB repository (http://dx.doi.org/10.15152/QDB.235 accessed on 21 June 2021.).

Structure-based design is directly impacted by the X-ray crystal protein structures and conformations of these that are employed. In the present work, it could be that the chimp AR structure best captures the conformation for binding actives/inactives, over those of rat and human.

The data set provided was challenging for any method, given the fact that the data is strongly unbalanced, containing a far larger number of non-actives than actives. This was true for the training set, *n*_active_ = 205, *n*_inactive_ = 1480; as well as for the evaluation set, *n*_active_ = 453, *n*_inactive_ = 3429. In addition, the protein structural information was not enough on its own to separate all the compounds. Though it could distinguish between binders and non-binders in the training set for those compounds that produced a docking score, there were a large number of compounds that had a docking score of 0, which required the use of additional techniques such as ligand-based ECFP fingerprints and statistical methods, such as Bayesians and combinations in consensus multivariate logistic regressions.

## 5. Conclusions

A variety of methods were systematically elaborated to model the binding of compounds to the androgen receptor for unbalanced data in order to help predict possible androgen pathway-disrupting compounds. The best methods out of the 13 tested included a multivariate logistic regression on values combining structure-based docking scores on the chimp protein, ligand-based Tanimoto dissimilarity distances using extended chemical fingerprints, and statistic comparisons between known binders and non-binders to the androgen receptor; as well as a multivariate logistic regression on docking scores and ECFP fingerprints. The best model includes only five descriptors: the docking score for the compound with chimp protein (ChimpDockScore), average of the distances to the known active (toxic) chemicals (avgD_Act_), average of the distances to the known inactive (non-toxic) chemicals (avgD_Inact_), the Bayesian probability value for the compound according to the distribution of known actives (toxic) compounds towards the chimp protein (P_Act_dockChimp_), and the Bayesian probability value for the compound according to the distribution of known inactive (non-toxic) compounds towards the chimp protein (P_Inact_). The model performed satisfactorily on the evaluation test provided in the CoMPARA project using MCC, BCR, and Acc. measures, as well as on an external reference set of compounds used in other studies and has easily interpretable variables and physicochemical reasoning.

## Figures and Tables

**Table 1:** True positives (TP), true negatives (TN), false positives (FP), false negatives (FN), specificity (SP, %), sensitivity (SE, %), accuracy (Acc., %), and Matthews correlation coefficient (MCC) for 13 different procedures for the training set.

Procedure		TP	TN	FP	FN	SP	SE	Acc.	MCC
**1**	Docking score threshold	97	1157	323	108	78.18	47.32	74.42	0.1926
**2**	Bayesian on scores	89	1232	248	116	83.24	43.41	78.40	0.2179
**3**	Logistic regression on Bayesian	100	1078	402	105	72.84	48.78	69.91	0.1545
**4**	Modified Bayesian	90	1233	247	115	83.31	43.90	78.52	0.2224
**5**	Fingerprints (ECFP)	189	795	685	16	53.72	92.20	58.40	0.3004
**6**	Docking scores then ECFP	169	978	502	36	66.08	82.44	68.07	0.3240
**7**	ECFP then docking scores	191	712	768	14	48.11	93.17	53.59	0.2725
**8**	Logistic regr. on docking scores and ECFP	71	1463	17	134	98.85	34.63	91.04	0.4920
**9**	Consensus Docking and ECFP else **8**	170	1054	426	35	71.22	82.93	72.64	0.3702
**10**	Consensus Bayesian and ECFP else **8**	117	1302	178	88	87.97	57.07	84.21	0.3875
**11**	Logistic regr. on docking scores and ECFP and ratio of Bayesian	69	1463	17	136	98.85	33.66	90.92	0.4829
**12**	Logistic regression on and Bayesian avgs. and fingerprints	42	1462	18	163	98.78	20.49	89.26	0.3400
**13**	Logistic regression on docking scores and Bayesian avgs. and fingerprints	75	1469	11	130	99.26	36.59	91.63	0.5324

**Table 2:** PPV (probability of predicted binder), NPV (probability of predicted nonbinder), positive (+LR) and negative (−LR) likelihood ratios, and BCR (modified correct classification rate) for 13 different procedures for the training set.

Procedure	NPV	PPV	+LR	−LR	BCR
**1**	91.4145	19.8795	2.1133	0.7999	0.2774
**2**	91.3944	22.6107	2.1079	0.6793	0.4356
**3**	91.1243	19.9203	1.7959	0.7032	0.4618
**4**	91.4562	26.7062	2.6270	0.6735	0.3855
**5**	91.8699	87.2093	49.2239	0.6389	0.2535
**6**	98.0271	21.6247	1.9920	0.1453	0.4488
**7**	96.4497	25.1863	2.4305	0.2657	0.6211
**8**	98.0716	19.9166	1.7955	0.1420	0.3881
**9**	91.6093	80.6818	30.1521	0.6613	0.2388
**10**	96.7860	28.5235	2.8810	0.2397	0.6805
**11**	93.6691	39.6610	4.7454	0.4880	0.5011
**12**	91.4947	80.2326	29.3027	0.6711	0.2306
**13**	89.9692	70.0000	16.8455	0.8049	0.1294

**Table 3:** AR pathway in vitro reference compounds and their predicted class according to Procedure **13**.

CAS	Name	Agonist	Antagonist	Predicted	Correct
52806-53-8	hydroxyflutamide	NA	Strong	0	X
90357-06-5	Bicalutamide	NA	Strong	0	X
122-14-5	Fenitrothion	NA	Strong	0	X
63612-50-0	Nilutamide	Negative	Moderate	0	X
427-51-0	cyproterone acetate	Weak	Moderate	1	Yes
80-05-7	bisphenol A	NA	Moderate/weak	1	Yes
330-55-2	Linuron	NA	Moderate/weak	0	X
13311-84-7	Flutamide	Negative	Moderate/weak	0	X
67747-09-5	Prochloraz	Negative	Moderate/weak	0	X
789-02-6	*o*,*p*′-DDT	Negative	Weak	0	Yes
60168-88-9	Fenarimol	Negative	Very weak	0	Yes
58-18-4	methyl testosterone	Strong	Negative	1	Yes
58-22-0	Testosterone	Strong	Negative	1	Yes
63-05-8	4-androstenedione	Moderate	Negative	1	Yes
1912-24-9	Atrazine	Negative	Negative	0	Yes
52918-63-5	Deltamethrin	Negative	Negative	0	Yes
10161-33-8	17b-trenbolone	Strong	NA	1	Yes
797-63-7	Levonorgestrel	Strong	NA	1	Yes
68-22-4	Norethindrone	Strong	NA	1	Yes
521-18-6	5a-dihydrotestosterone	Strong	NA	1	Yes

## Data Availability

The data and best model presented in this study are openly available at QsarDB Repository at http://dx.doi.org/10.15152/QDB.235, accessed on 21 June 2021.

## Supplementary Materials

The following are available online at https://www.mdpi.com/article/10.3390/ijms22136695/s1.
